# Growth Properties and Metabolomic Analysis Provide Insight into Drought Tolerance in Barley (*Hordeum vulgare* L.)

**DOI:** 10.3390/ijms25137224

**Published:** 2024-06-29

**Authors:** Juncheng Wang, Lirong Yao, Jing Hao, Chengdao Li, Baochun Li, Yaxiong Meng, Xiaole Ma, Erjing Si, Ke Yang, Hong Zhang, Xunwu Shang, Huajun Wang

**Affiliations:** 1 State Key Laboratory of Aridland Crop Science, Gansu Key Lab of Crop Improvement and Germplasm Enhancement, Lanzhou 730070, China; wangjc@gsau.edu.cn (J.W.); yaolr@gsau.edu.cn (L.Y.); haoj@gsau.edu.cn (J.H.); libc@gsau.edu.cn (B.L.); mengyx@gsau.edu.cn (Y.M.); maxl@gsau.edu.cn (X.M.); siej@gsau.edu.cn (E.S.); yangk@gsau.edu.cn (K.Y.); zhangh@gsau.edu.cn (H.Z.); 2 Department of Crop Genetics and Breeding, College of Agronomy, Gansu Agricultural University, Lanzhou 730070, China; 15293153957@163.com; 3 Western Barley Genetics Alliance, College of Science, Health, Engineering and Education, Murdoch University, Murdoch, WA 66667, Australia; c.li@murdoch.edu.au; 4 Department of Botany, College of Life Sciences and Technology, Gansu Agricultural University, Lanzhou 730070, China

**Keywords:** bioprocesses, cereal crop, drought stress, expression patterns, metabolomic regulation

## Abstract

Drought stress is a major meteorological threat to crop growth and yield. Barley (*Hordeum vulgare* L.) is a vital cereal crop with strong drought tolerance worldwide. However, the underlying growth properties and metabolomic regulatory module of drought tolerance remains less known. Here, we investigated the plant height, spike length, effective tiller, biomass, average spikelets, 1000-grain weight, number of seeds per plant, grain weight per plant, ash content, protein content, starch content, cellulose content, and metabolomic regulation mechanisms of drought stress in barley. Our results revealed that the growth properties were different between ZDM5430 and IL-12 under drought stress at different growth stages. We found that a total of 12,235 metabolites were identified in two barley genotype root samples with drought treatment. More than 50% of these metabolites showed significant differences between the ZDM5430 and IL-12 roots. The Kyoto Encyclopedia of Genes and Genomes pathway analysis identified 368 differential metabolites mainly involved in starch and sucrose metabolism, the pentose phosphate pathway, pyrimidine metabolism, phenylalanine, tyrosine, and tryptophan biosynthesis in ZDM5430 under drought stress, whereas the different metabolites of IL-12 under drought stress related to starch and sucrose metabolism, the pentose phosphate pathway, 2-oxocarboxylic acid metabolism, cutin, suberine and wax biosynthesis, carbon metabolism, fatty acid biosynthesis, and C5-branched dibasic acid metabolism. These metabolites have application in the tricarboxylic cycle, the urea cycle, the met salvage pathway, amino acid metabolism, unsaturated fatty acid biosynthesis, phenolic metabolism, and glycolysis. On the other hand, the expression patterns of 13 genes related to the abovementioned bioprocesses in different barley genotypes roots were proposed. These findings afford an overview for the understanding of barley roots’ metabolic changes in the drought defense mechanism by revealing the differently accumulated compounds.

## 1. Introduction

Drought stress is a significant abiotic stress that limits the productivity and distribution of crops worldwide [1]. Mild to moderate drought stress (relative leaf water content down to 70%) leads to an osmotic stress, ionic imbalance, and nutrient deficiency in plants [2,3]. Improving the drought tolerance of crops and managing the drought stress are necessary to meet increasing food supply demands [4]. However, coping with drought stress involves complicated mechanisms that include morphological, physiological, developmental, and biochemical strategies [5]. Furthermore, drought stress regulation metabolites lead to changes in the metabolite profiling that help plants to adapt to drought environments [6]. Many drought-responsive metabolite system, which are involved in sugar synthesis, photosynthesis, glycolysis, the tricarboxylic acid (TCA) cycle, hormone synthesis, and amino acids, play important roles under drought stress conditions [7]. Previous research has suggested that organic acids, amino acids, sugars, and low molecular weight compounds increased in root samples of *Triticeae* species under drought stress; ferulic acid and 4-hydroxycinnamic acid were also thought to be vital metabolites for drought tolerance [8,9]. The key responsive compounds in leaves under drought stress were amino acid metabolomic changes, as described by Chmielewska et al. [10]. Metabolomics, a newly developing molecular biotechnology of the simultaneous and comprehensive analysis of organic molecular compound (metabolites) profiles, has been routinely applied in plants, animals, humans, and bacteria [11]. This is also a vital strategy to explicate plant adaptation and growth mechanisms under harsh environments, and it has been advocated when understanding how plants cope with abiotic stresses. 

Barley (*Hordeum vulgare* L.) is one of the most important food sources, livestock feeds, and raw materials for brewing and malting worldwide [4]. Focusing on drought, it is a major threat to the stable yield of barley [12]. Recently, the physical, genetic, and functional sequence on the barley genome has been assembled (The International Barley Genome and Sequencing Consortium, 2012) [13]. Drought-tolerant and susceptible barley varieties were also lately examined under abiotic stresses and subjected to metabolomic analyses [14]. However, there is a lack of publications that simultaneously covers the agronomic, quality traits, and metabolomic research performed in one analysis in different barley genotypes. For this research, we analyzed the plant height, spike length, effective tiller, biomass, average spikelets, 1000-grain weight, number of seeds per plant, grain weight per plant, ash content, protein content, starch content, and cellulose content of two barley genotypes under drought stress levels at different growth stages using physiological approaches. We also investigated the metabolome changes in roots of different barley genotypes subjected to drought. The results investigated at the harvest stage revealed significant differences in the tolerance to drought stress between those two varieties. 

## 2. Results

### 2.1. Effect of Drought Stress on Agronomic and Quality Traits of Barley

**Figure 1:**
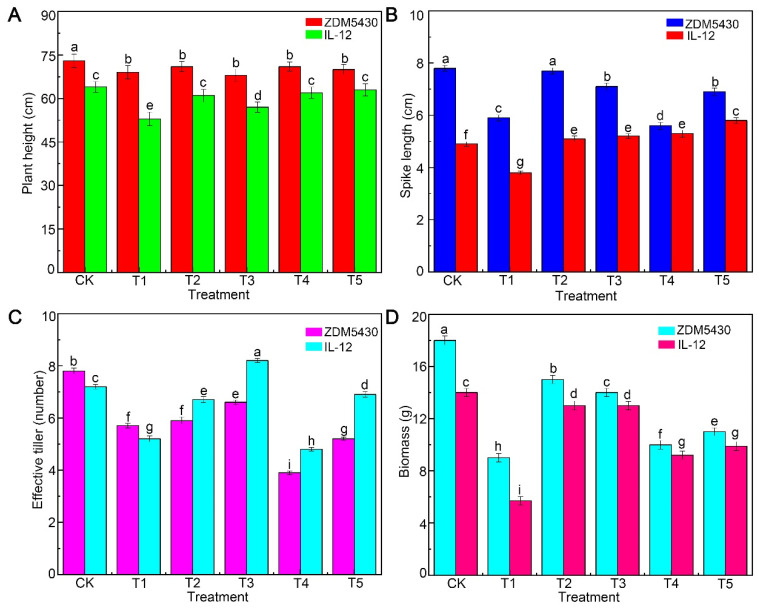
Effects of drought stress on plant height (**A**), spike length (**B**), effective tiller (**C**), and biomass (**D**) of barley at different growth stages. Data are shown as mean ± standard error (n = 3). ANOVA: Since two-way ANOVA revealed a significant ozone effect on PII values according to Tukey’s post hoc test, different letters indicate significant differences among means in each group (*p* ≤ 0.05).

**Figure 2:**
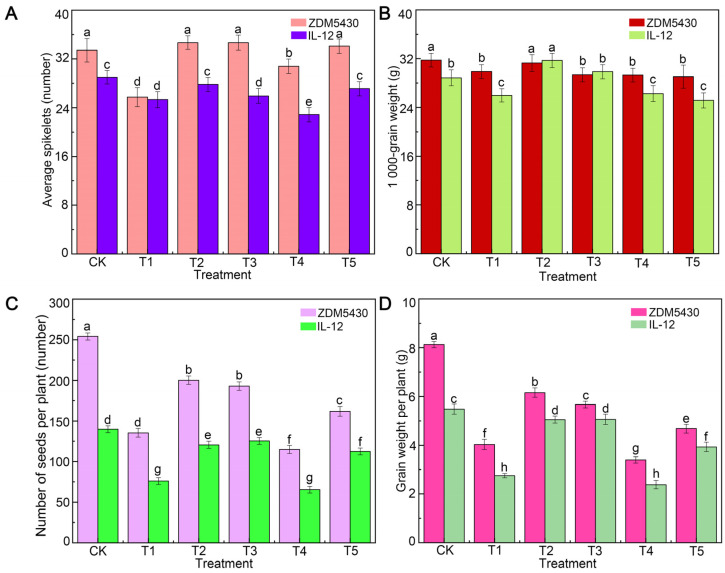
Effects of drought stress on average spikelets (**A**), 1000-grain weight (**B**), number of seeds per plant (**C**), and grain weight per plant (**D**) of barley at different growth stages. Data are shown as mean ± standard error (n = 3). ANOVA: Since two-way ANOVA revealed a significant ozone effect on PII values according to Tukey’s post hoc test, different letters indicate significant differences among means in each group (*p* ≤ 0.05).

**Figure 3:**
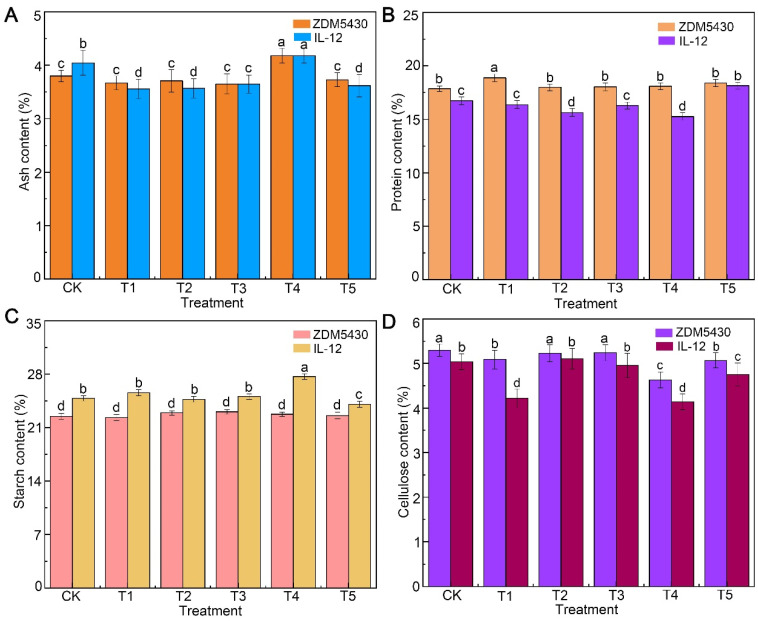
Effects of drought stress on ash content (**A**), protein content (**B**), starch content (**C**), and cellulose content (**D**) of barley at different growth stages. Data are shown as mean ± standard error (n = 3). ANOVA: Since two-way ANOVA revealed a significant ozone effect on PII values according to Tukey’s post hoc test, different letters indicate significant differences among means in each group (*p* ≤ 0.05).

The agronomic and quality traits of ZDM5430 and IL-12 under different drought conditions were analyzed (Figure 1, Figure 2 and Figure 3). Under T1 treatment, the obvious change was found in the plant height, spike length, and biomass between the two barley genotypes. The spike length, average spikelets, and 1000-grain weight showed no decrease in ZDM5430 under the T2 condition. And, ZDM5430 had a significant advantage in terms of plant height, average spikelets, and 1000-grain weight under drought stress. However, the previously described agronomic traits showed a significant decrease in IL-12 under different treatments (Figure 1 and Figure 2).

As for ZDM5430, the protein content was 1.22% higher than that of the control group, and the contents of ash, starch, and cellulose decreased under the T1 condition. When compared with the control, the contents of ash, protein, starch, and cellulose showed no significant difference in the T2 condition, as well as in the T3 condition. In addition, the contents of ash, protein, and cellulose in ZDM5430 were higher than that in IL-12 under each treatment. And, the starch content changed a little in the two barley genotypes under drought stresses (Figure 3). 

### 2.2. Effect of Different Drought Stress Levels on Grain Morphology and Starch Structure Features

**Figure 4:**
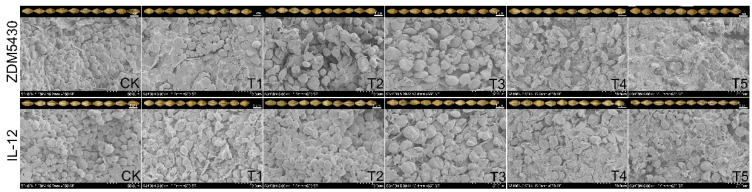
Ultrastructure starch granules of seed in the drought-tolerant barley genotype and drought-sensitive barley genotype under drought stress during the entire growth stage (T1), the seedling stage (T2), the jointing stage (T3), the heading stage (T4), and the grain filling stage (T5).

The grain morphological traits consisting of grain length, width, width-to-length ratio, and volume showed a marked variation under different drought stresses in ZDM5430 and IL-12 compared to the CK group. However, the grain length in ZDM5430 was higher than that of IL-12, and the grain volume displayed the opposite situation under their respective treatment. Compared to the control, the width-to-length ratio of IL-12 changed little under different drought stresses (Table 1). The size of the starch structure increased, and the numbers of small starch granules decreased in the two barley genotypes between the treatments T1, T2, T3, and the control. But, the numbers of starch granules significantly decreased under the T4 and T5 conditions in comparison to their own contrast, especially in ZDM5430 (Figure 4). 

### 2.3. Phenotypic and Physiological Responses and Metabolic Profiles of Two Barley Genotypes under Drought Stress

**Figure 5:**
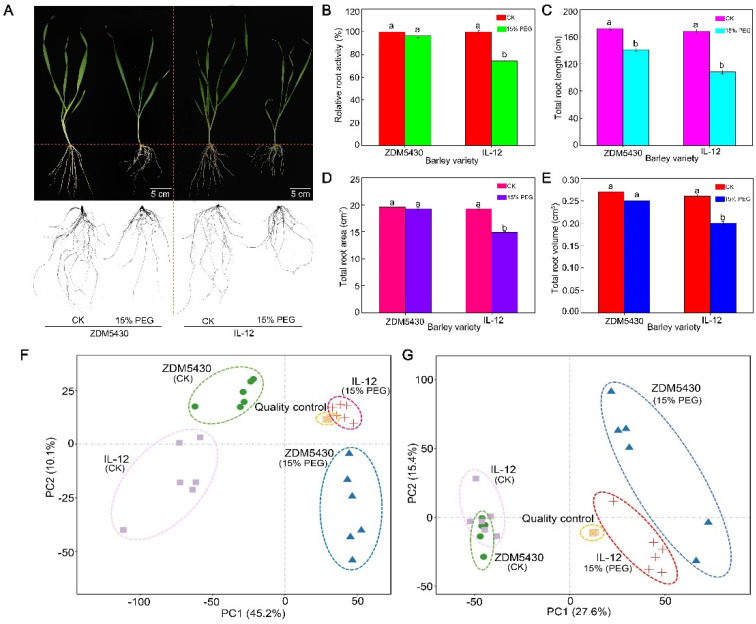
(**A**) Phenotype, (**B**) relative root activity, (**C**) total root length, (**D**) total root area, and (**E**) total root volume in ZDM5430 and IL-12, as well as overall score plots of the principal component analysis (PCA) model with quality control in (**F**) positive and (**G**) negative ionization modes. PC1—first principal component; PC2—second principal component; PEG—polyethylene glycol 6000. Data are shown as mean ± standard error (n = 3). ANOVA: Since two-way ANOVA revealed a significant ozone effect on PII values according to Tukey’s post hoc test, different letters indicate significant differences among means in each group (*p* ≤ 0.05).

As shown in Figure 5A, drought stress resulted in an inhibition of root growth. Compared to the control, the relative root activity, total root length, total root area, and total root volume in IL-12 showed a reduction of 79.32%, 61.11%, 78.42%, and 75.55%, respectively (Figure 5B–E; *p* < 0.05 for all). But, no significant difference was noted in the relative root activity, total root area, and total root volume in ZDM5430. Thus, these physiological features indicated that the ZDM5430 spring barley genotype displayed higher drought tolerance than the IL-12 barely genotype.

We used PCA to monitor the reproducibility and accuracy of the analysis process of ZDM5430 and IL-12 roots under control conditions and drought stress, as well as QC samples. Biological replicates of every sample were grouped together using small confidence intervals. The first principal component (PC1) and the second principal component (PC2) explained 45.2% and 10.1%, respectively, of the total variability for positive ion mode (POS) datasets(Figure 5F). Similarly, PC1 and PC2 explained 27.6% and 15.4%, respectively, of the total variability for negative ion mode (NEG) datasets(Figure 5G). For both ion modes, the metabolic profiles of ZDM5430 and IL-12 roots were separated distinctly into PC1 and PC2.

### 2.4. Qualitative and Quantitative Metabolites

A total of 12,235 metabolites were identified using a widely targeted metabolites method, of which 5,585 and 6,650 metabolites were for POS and NEG, respectively (Tables S1 and S2). There were 1,710 known metabolites, and it was important to note that 10,525 unknown metabolites were determined.

**Figure 6:**
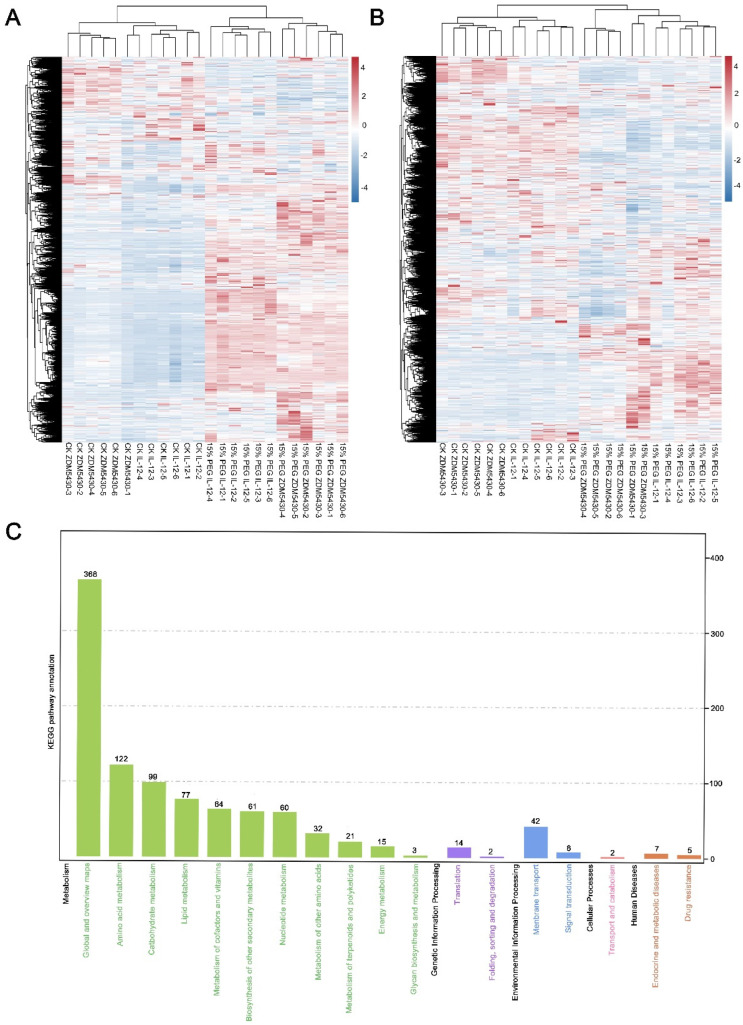
(**A**,**B**) Hierarchical cluster description and (**C**) KEGG pathway enrichment description of all identified metabolites. The heat map presents the expression of differential metabolites between ZDM5430 and IL-12 in (**A**) positive and (**B**) negative ionization modes. Red represents up-regulated metabolites, and blue color shows downregulated metabolites based on a log2-fold change expression value. KEGG—Kyoto Encyclopedia of Genes and Genomes.

Hierarchical cluster analysis was used to estimate differences in the metabolite accumulation patterns between different barley root samples. The sample classes were separated into four nonoverlapping clusters, depending on the varieties (Figure 6A,B), thus indicating that the metabolite accumulation was genotype-specific (www.omicsmart.com, accessed on 17 March 2024). The metabolite production was mainly enriched in global and overview maps (368 metabolites), amino acid metabolism (122 metabolites), carbohydrate metabolism (99 metabolites), lipid metabolism (77 metabolites), metabolism of cofactors and vitamins (64 metabolites), biosynthesis of other secondary metabolites (61 metabolites), and nucleotide metabolism (60 metabolites; Figure 6C).

### 2.5. Different Metabolite Identification between Varieties

The PCA indicated that these sample metabolites were well separated along the PC1; thus, the variety-specific metabolism of barley was clear (Figures S1 and S2; Tables S3–S10). Compared to the PCA, the OPLS-DA can increase the distinction between each treatment and is more beneficial in determining differential metabolites based on incorporating an orthogonal signal correction filter into a PLS model. Here, the values of R2 and Q2 exceeded 0.9 for CK ZDM5430 vs. CK IL-12, CK ZDM5430 vs. 15% PEG ZDM5430, CK IL-12 vs. 15% PEG IL-12, and 15% PEG ZDM5430 vs. 15% PEG ZDM5430. In addition, the OPLS-DA model was identified through permutation tests using 100 alignment tests. The R2’ and Q2’ values of replacement were less than the corresponding values of R2 and Q2 in the original model (Figures S3 and S4; Tables S3–S10). These results show that the model may be used to select important metabolites in ≥ 1 and a *t* test *p* < 0.05 between genotypes.

### 2.6. Analysis of Differential Metabolites between Genotypes

**Figure 7:**
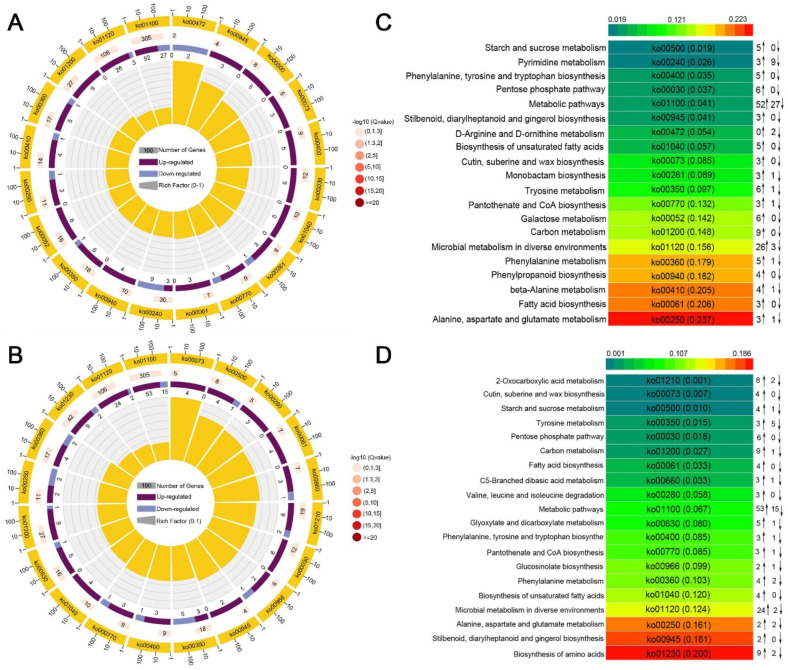
KEGG pathway enrichment analysis of differential metabolites in ZDM5430 and IL-12 under drought stress. (**A**) KEGG pathway enrichment analysis of differential metabolites in CK ZDM5430 vs. 15% PEG ZDM5430. (**B**) KEGG pathway enrichment analysis of differential metabolites in CK IL-12 vs. 15% PEG IL-12. (**C**) The top 20 of KEGG pathway enrichment in ZDM5430 under drought stress. (**D**) The top 20 of KEGG pathway enrichment in IL-12 under drought stress. KEGG—Kyoto Encyclopedia of Genes and Genomes; PEG—polyethylene glycol 6000; CK—normal treatment; 15% PEG; 15% PEG 6000 treatment. Up arrow—up expression; down arrow—down expression.

A KEGG pathway enrichment analysis with different genotypes of barley at *p* < 0.05 was performed to study the key pathways (Figure 7). Under drought stress, different metabolites in the drought-tolerant genotype ZDM5430 were mainly involved in starch and sucrose metabolism, pyrimidine metabolism, phenylalanine, tyrosine and tryptophan biosynthesis, the pentose phosphate pathway, metabolic pathways and stilbenoid, and diarylheptanoid and gingerol biosynthesis. In comparison, the different metabolites of IL-12 treated with drought stress were mainly involved in 2-oxocarboxylic acid metabolism; cutin, suberine, and wax biosynthesis; starch and sucrose metabolism; tyrosine metabolism; the pentose phosphate pathway; carbon metabolism; fatty acid biosynthesis; and C5-branched dibasic acid metabolism. We noticed that the pathways of the starch and sucrose metabolism and the pentose phosphate pathway were commonly found in the two genotypes treated with drought stress. However, the starch and sucrose metabolism pathways were the most significant enrichment in the drought-tolerant genotype ZDM5430, and the 2-oxocarboxylic acid metabolism pathway was the most significant enrichment in IL-12 drought-sensitive genotype. These results suggest that the accumulation of the same metabolic pathway and/or the enrichment of specific metabolic pathways lead to differences in drought tolerance between the two cultivars. 

### 2.7. Comprehensive Metabolic Networks Analysis under Drought Stress

To comprehensively understand the metabolite changes in the two genotypes under drought stress, we proposed a metabolic pathway according to Zhao et al. [15] and a web-based database of metabolic pathways. The important known pathways contain the TCA cycle, the urea cycle, the met salvage pathway, amino acid metabolism, unsaturated fatty acid biosynthesis, phenolic metabolism, glycolysis, and glutamate-mediated proline biosynthesis.

**Figure 8:**
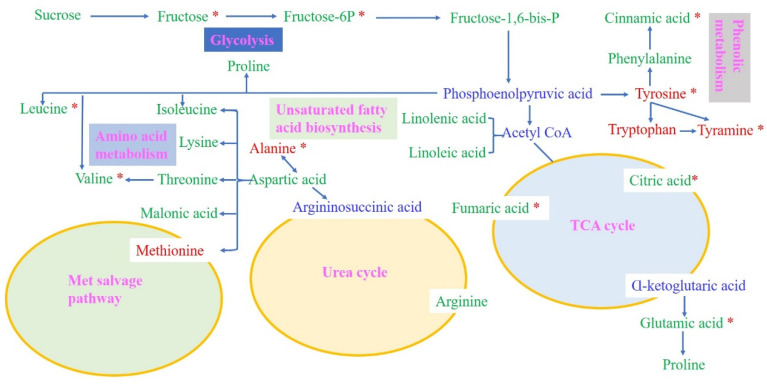
Analysis of metabolic networks in the roots of ZDM5430 under drought stress. The metabolic pathways were derived from the literature and a web-based database of metabolic pathways. The metabolites in blue text were not identified in this research. Differential metabolite changes were detected by a log_2_ ratio. Red indicates an increase in level, and green indicates a decrease in level. * represents a significant difference. Blue indicates no detection in level.

For the ZDM5430 drought-tolerant genotype, we identified five metabolites related to the glycolysis pathway, such as sucrose, fructose, fructose-6-phosphate, fructose-1,6-bisphosphate, and sorbitol. Under drought stress, fructose, fructose-6-phosphate, fructose-1,6-bisphosphate, and sorbitol significantly decreased. Phosphoenolpyruvic acid became converted to shikimate and acetyl coenzyme A separately. However, acetyl coenzyme A entered the TCA cycle. The metabolites involved in the TCA cycle were citric acid and fumaric acid, which revealed a significant decrease under drought stress. Pyroglutamic acid, proline, and aminobutyric acid are biosynthesized through the glutamate-mediated pathway from α-ketoglutaric acid. Aminobutyric acid appeared to significantly increase under drought stress, thus suggesting that aminobutyric acid can be effectively stimulated in ZDM5430 in response to drought stress. Shikimate can become converted to phenylalanine, tryptophan, and tyrosine, thus becoming incorporated into phenolic metabolism. The phenylalanine content increased, and the tyrosine content significantly increased for samples treated with drought stress. Similarly, the tryptophan content decreased, and the cinnamic acid content significantly decreased under drought stress. In the urea cycle, the ornithine content increased, and the arginine content significantly increased. In addition, met salvage pathway metabolites, amino acid metabolism, and unsaturated fatty acid biosynthesis were also identified to be involved in the response to drought. Of these, the nicotinic acid content and linolenic acid content significantly increased, and the malonic acid content significantly decreased under drought stress (Figure 8).

**Figure 9:**
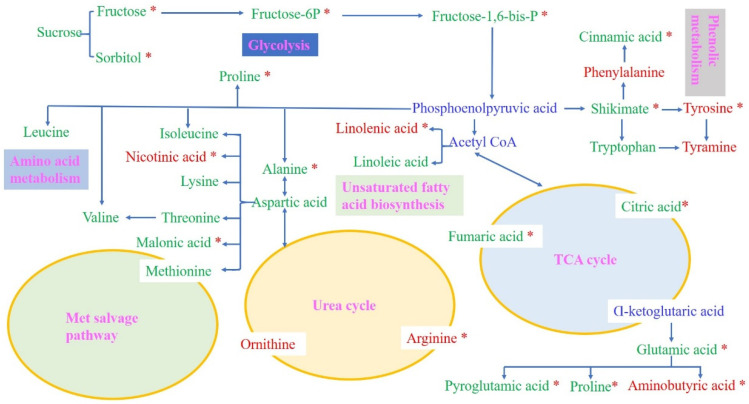
Analysis of metabolic networks in the roots of IL-12 under drought stress. The metabolic pathways were derived from the literature and a web-based database of metabolic pathways. The metabolites in blue text were not identified in this research. Differential metabolite changes were detected by a log_2_ ratio. Red indicates an increase in level, and green indicates a decrease in level. * represents a significant difference. Blue indicates no detection in level.

However, for the IL-12 drought-sensitive genotype, four metabolites were involved in the glycolysis pathway, including sucrose, fructose, fructose-6-phosphate, and fructose-1,6-bisphosphate. Under drought stress, these metabolite levels displayed decreases, and the fructose and fructose-6-phosphate levels were significantly decreased. In contrast, the tyrosine, tyramine, and tryptophan levels showed increases, and the tyrosine and tyramine levels significantly increased under phenolic metabolism. The metabolites involved in the TCA cycle were citric acid, fumaric acid, and glutamic acid, which showed a significant decrease under drought stress. Proline was biosynthesized by the glutamate-mediated pathway from α-ketoglutaric acid; the proline level also decreased. Similarly, the levels of valine and leucine significantly decreased, and the levels of isoleucine, lysine, threonine, and malonic acid revealed decreases under amino acid metabolism. Linolenic acid and linoleic acid also decreased in unsaturated fatty acid biosynthesis. These findings indicated that drought stress inhibited the metabolism of the IL-12 drought-sensitive genotype (Figure 9).

### 2.8. qRT-PCR Analysis Elucidated That Drought Treatment Orchestrated the Expression of Genes Involved in Metabolic Networks

**Figure 10:**
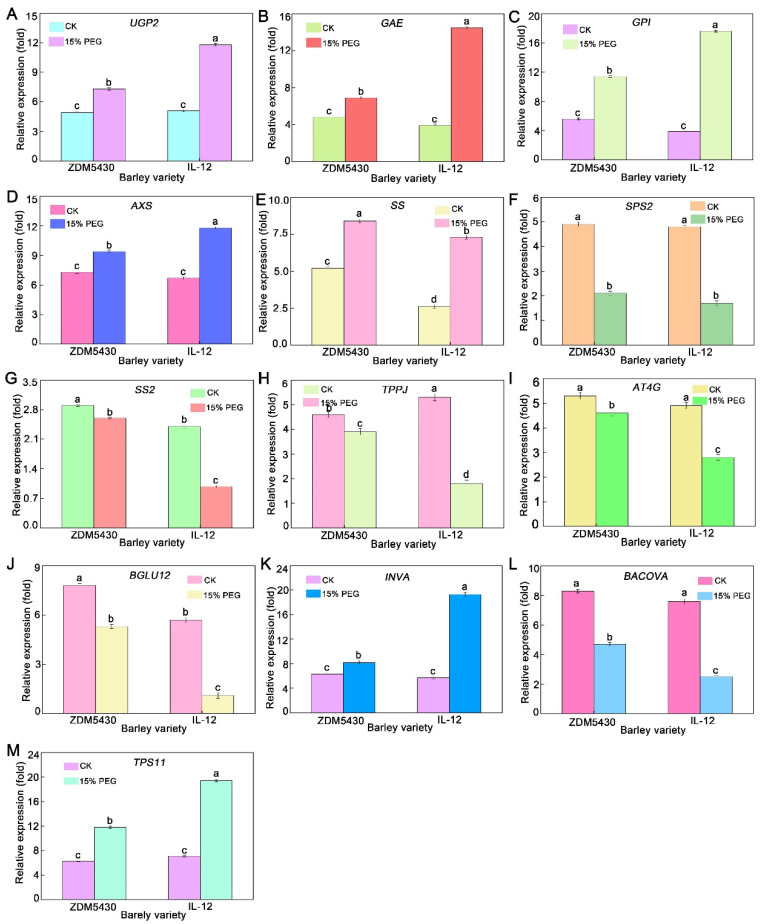
Effects of drought stress on the transcriptional levels and expression kinetics of starch and sucrose metabolism genes in barley root. ((**A**) UGP2: Uridine diphosphate glucose pyrophosphorylase; (**B**) GAE: Uridine glucuronic acid 4 epimerase; (**C**) GPI: Glucose 6-phosphate isomerase; (**D**) AXS: Uridine celery sugar/xylose synthase; (**E**) SS: sucrose synthase; (**F**) SPS2: sucrose phosphate synthase; (**G**) SS2: starch synthase; (**H**) TPPJ: Trehalose 6 Phosphatase; (**I**) AT4G: endoglucanase; (**J**) BGLU12: glucosidase; (**K**) INVA: β fructofuranosidase; (**L**) BACOVA: β glucosidase; (**M**) TPS11: Trehalose 6 phosphate synthase). Data are shown as mean ± standard error (n = 3). ANOVA: Since two-way ANOVA revealed a significant ozone effect on PII values according to Tukey’s post hoc test, different letters indicate significant differences among means in each group (*p* ≤ 0.05).

To verify the gene expressions in relation to the metabolic networks of barley roots after drought treatment, qRT-PCR analysis was performed on 13 selected genes mainly involved in the described metabolic pathway (Figure 10). The expressions of the genes Uridine diphosphate glucose pyrophosphorylase (*UGP2*), Uridine glucuronic acid 4 epimerase (*GAE*), Glucose 6-phosphate isomerase (*GPI*), Uridine celery sugar/xylose synthase (*AXS*), sucrose synthase (*SS*), β fructofuranosidase (*INVA*), and Trehalose 6 phosphate synthase (*TPS11*) increased, and the expression patterns of sucrose phosphate synthase (*SPS2*), starch synthase (*SS2*), Trehalose 6 Phosphatase (*TPPJ*), endoglucanase (*ATG4*), β glucosidase (*BACOVA*), and glucosidase (*BGLU12*) decreased in two barley roots treated with 15% PEG 6000 for 21 days. However, the levels of *UGP2*, *GAE*, *GPI*, *AXS*, *INVA*, and *TPS11* increased more obviously in IL-12 than those in ZDM5430; they were much lower for *SS2*, *TPPJ*, *ATG4*, *BGLU12*, and *BACOVA* in IL-12 than those in ZDM5430 under drought stress.

## 3. Discussion

### 3.1. Growth of Barley under Different Drought Conditions

Drought stress is an important abiotic stress that can influence plant emergence, leaf, root, tiller and stem development and growth, dry matter production, floral initiation, pollination, and seed yield and quality [16,17]. Meanwhile, it is important to understand that the negative effects during one phase or trait could be compensated by the recovery of the other organ or trait. For example, lower emergence can often be compensated through increasing tillering or branching, or the greater seed numbers could be hindered by poor grain or partially filled seeds [17]. In our study, compared to the control, the spike length, effective tiller, biomass, number of seeds per plant, grain weight per plant, and grain size were negatively affected by different drought stresses in ZDM5430, but the plant height, average spikelets, and 1000-grain weight could not display significant differences. For IL-12, the plant height, biomass, number of seeds per plant, grain weight per plant, and grain size showed significantly decreases, the spike length increased, and the 1000-grain weight could not display a significant difference under different drought stresses. In addition, the T2 treatment could not cause a significant difference for the spike length, average spikelets, 1000-grain weight, grain width, and grain width-to-length ratio for ZDM5430. Drought stress can have a profound impact on the seed quality of cereals mainly because of its impact on nutrient uptake, assimilate supply, partitioning, and the remobilization of nutrients. The impacts on nutritional quality have mainly been discussed in terms of starch, ash, cellulous, and protein contents [18,19]. Furthermore, drought influence on starch granules size may lead to changes in overall seed growth rates, which in turn are likely to be based on the carbon and nitrogen supply to the seeds [20]. ZDM5430 showed the higher content in ash, protein, and cellulose than that of IL-12 under the individual drought stress; the starch content showed the opposite results. Compared with their respective contrast, the size of the starch structure increased, and the numbers of small starch granules decreased for ZDM5430 and IL-12 under the T1, T2, and T3 conditions. But, the numbers of starch granules significantly decreased under the T4 and T5 conditions in comparison with the contrast, especially in ZDM5430. The results indicate the growth exhibited for the drought-tolerant barley genotype and the drought-sensitive barley genotype under different drought stresses conditions.

### 3.2. Root Growth of ZDM5430 and IL-12 under Drought Stress

Drought stress usually restrains shoot growth but stimulates root growth to promote the remobilization of photoassimilates from shoots to roots for survival [21]. In wheat, the number of roots in drought-tolerant varieties was fewer in the surface soil. However, a dense root architecture was apparent in the subsoil layer under drought stress, which allows for penetration into the deep soil to absorb water [22]. The wheat genotype with a greater root length density and root mass had enhanced access to subsoil water after anthesis, thus contributing to a high grain yield when soil water was scarce [22,23]. In our study, the relative root activity, total root length, total root area, and total root volume in the drought-tolerant ZDM5430 genotype were all higher than those in the drought-sensitive IL-12 genotype under drought stress. Moreover, drought stress had little effect on the relative root activity, total root area, or total root volume of ZDM5430, but these were significantly inhibited in IL-12 under drought stress. Obviously, the degree of drought injury between different barley genotypes differed; ZDM5430 should be more suitable for a dry area with lower irrigation and showed higher yield stability. 

### 3.3. Amino Acids Change in Response to Drought Stress

It is well known that amino acids are the best nitrogen source for plants. Many reports have described how specific amino acids may defer protein degradation under drought stress [24]. The significant enrichment of amino acids and derivatives protects the plant’s defense from drought stress through osmotic balancing and sustaining the stability of the cytoplasmic membrane [25]. Guo et al. found that the concentration of 16 amino acids and their derivatives (arginine, threonine, asparagine, glycine, valine, isoleucine, tyrosine, alanine, and leucine) increased in response to drought stress within 6 to 10 days [26]. Naimat et al. identified how amino acids and their derivatives increased in root samples of Triticeae species under drought stress [9]. Vian et al. detected 132/141 amino acid metabolites that were significantly enriched in drought stress under developing maize kernels [27]. The branched chain amino (leucine, isoleucine, and valine), proline, tryptophan, and histidine were commonly observed to respond to drought in plants [28]. Our study showed that the metabolites of aminobutyric acid, arginine, nicotinic acid, linolenic acid, and tyrosine showed a significant increase, as well as that the accumulation of citric acid, glutamic acid, fumaric acid, alanine, malonic acid, and cinnamic acid were significantly decreased in the drought-tolerant ZDM5430 genotype under drought stress. Additionally, alanine, tyrosine, and tyramine metabolites showed a significant increase, and citric acid, glutamic acid, fumaric acid, leucine, valine, and cinnamic acid enrichment significantly decreased in the IL-12 drought-sensitive genotype under drought stress. The above indicates that amino acids and their derivatives are the important modulated factors in response to drought stress in crops.

### 3.4. Proline and Phenylalanine Changes in Response to Drought Stress

Proline is a type of osmotic regulator that is considered to enhance plant tolerance and regulate the cell’s defense from various abiotic stresses [29]. Chmielewska et al. described changes in metabolites in barley and identified proline accumulation in the leaves of barley genotypes subjected to drought [10]. Yang et al. analyzed metabolites related to a drought response in maize and pointed out the higher levels of proline under drought conditions [30]. Proline was significantly accumulated in the common wild soybean, especially in drought-tolerant wild soybean (*p* < 0.01) [31]. Drought stress increased the proline content in young seedlings of wheat under drought stress after metabolite analysis; it was twice as high in T2 seedlings (20 d) than that in T1 seedlings (10 d) [32]. In contrast, proline accumulation was decreased in the roots of different barley genotypes, especially in drought-tolerant barley roots in our study. This implies that proline has a special role in barley roots subjected to drought stress.

Phenylalanine is a precursor of numerous important secondary metabolite pathways, including anthocyanins and flavonoids, and can affect cellular osmotic balance, as well as improve plant drought tolerance [33,34]. Rabara et al. [31] found that the aromatic amino acid, phenylalanine, significantly accumulated in drought-tolerant wild soybean. Sicher et al. reported significant phenylalanine accumulation in poplar plants under drought-induced conditions [24]. The higher levels of phenylalanine induced by drought indicate that this amino acid is important for the adaptation of Jerusalem artichokes to drought stress [15]. We also found an increased level of phenylalanine in the drought-tolerant ZDM5430 genotype under drought stress, while it was decreased for the drought-sensitive IL-12 genotype. Flavonoids, which are important secondary metabolites in plants, possess many functions in plant growth and are adapted to biotic and abiotic stresses [6]. The accumulation of flavonoids was a vital factor that increased in drought stress in Arabidopsis; caffeic acid and cinnamic acid significantly increased under drought stress, thus highlighting the effect of flavonoids for the plant in response to drought stresses [35]. However, we found that the level of cinnamic acid significantly decreased in both ZDM5430 and IL-12, which may play a special role in enhancing the ability of barley to avoid toxins through cell osmotic regulation under drought stress.

### 3.5. qRT-PCR Analysis Elucidated That Drought Treatment Orchestrated the Expression of Genes Involved in Starch and Sucrose Metabolism

In previous research, most of the genes involved in carbohydrate metabolism were shown to be simultaneously upregulated in transcriptional expression under drought stress [36]. Moreover, the genes involved in the starch and sucrose biosynthetic pathway also appeared to have high transcriptional expression levels [37]. The increased expression of genes, including *UPG2*, *GAE*, *GPl*, *AXS*, *SS*, *INVA*, and *TPS11*, were observed 21 days after drought treatment. However, the expressions of *SPS2*, *SS2*, *TPPJ*, *AT4G*, *BGLU12*, and *BACOVA* showed downregulation in barley roots treated with 15% PEG 6000 for 21 days. UGP2 is a key enzyme in polysaccharide biosynthesis: it can interact with SS and SPS to regulate the synthesis and metabolism of sucrose in plants [38]. In the starch and sucrose metabolism pathway, the sucrose content synthesized by photosynthesis is closely related to SS and SPS, which can further decompose sucrose and also affect the plant abiotic stress response [39]. Of particular note is the higher expression of *SS* in the root of ZDM5430 under drought stress, which participated in the initial carbohydrate metabolism and the encoding of the SS carrier protein as an important component in starch and sucrose biosynthesis. The genes *INVA* and *TPS11* of sugar 6-phosphate synthase and Trehalose 6-phosphate synthase are upregulated in expression in β-Furanofructosidase, which can convert sucrose into essential sugars, alcohols, and phenols for plant growth; Trehalose 6-phosphate synthase are synthesized and accumulated extensively in plants under drought stress, thereby stabilizing the cell structure and macromolecular activity such as proteins and nucleic acids, which can enhance plant drought resistance [40]. The upregulation of *INVA* and *TPS11* indicates that drought stress promotes starch degradation and soluble sugar accumulation, thereby regulating carbon flow in cells and providing energy for plants to tolerate drought, as well as enhancing barley tolerance to drought stress. More importantly, the expressions of *INVA* and *TPS11* were higher in IL-12 than those in ZDM5430 under 15% PEG 6000 stress, which indicates that the drought-tolerant barley was less affected by drought stress compared to the drought-sensitive material. 

Under drought stress, the expression of the *SPS2* gene in potatoes affects the activity of sucrose phosphate synthase and the amylose generation: an increase in the *SPS2* gene expression levels will enhance the antisense expression of ADP glucose, thereby inhibiting potato starch synthesis [41]. In this study, the downregulation of *SPS2* gene expression under drought stress indirectly affected the starch content, thus indicating that barley roots survive from drought stress based on the promotion of starch synthesis by downregulating the expression of the *SPS2* gene. 

With the increasing drought stress levels, the content of osmoregulation substances such as proline and soluble protein were found to increase to different degrees, while the content of starch was found to decrease to maintain its growth and development for the plant [42]. In this study, the genes of *TPPJ, AT4G, BLU12*, and *BACOVA* related to the starch and sucrose metabolism pathways were downregulated under drought stress, thus indicating that drought stress affects the sugar metabolism process of barley, and the effect on the water-soluble carbohydrates metabolism of IL-12 roots was more apparent. This is consistent with the transcriptional data on potatoes under drought stress. However, the mechanism of these genes/proteins participating in these metabolism pathways of barley roots under drought stress needs further study.

## 4. Material and Methods

### 4.1. Plant Growth and Drought Treatments

Two spring barley genotypes, ZDM5430 (drought-tolerant) and IL-12 (drought-sensitive), were used in this study, which were obtained from Gansu Agricultural University (Lanzhou, China). Seeds were sown in plastic pots (25 cm in height and 30 cm in diameter) filled with a mixture of nutrient soil and vermiculite (2:1 v/v). Plants were cultured in a rain-proof shelter watered with Hoagland solution, with 6 seeds per pot. Before drought treatment, the seedlings grew uniformly, and the soil moisture was sufficient to ensure consistency in the experiment. The seedlings were treated with drought stress when it grew to 4–5 leaves stage. This experiment consists of five treatments: drought stress throughout the entire growth stage (T1), drought stress during the seedling stage (T2), drought stress during the elongate stage (T3), drought stress during the heading stage (T4), and drought stress during the grain filling stage (T5). The control group was irrigated normally at each growth stage, and the soil average moisture content was 85.00% ± 1.21%. The treatment group was controlled to decrease to 45.00% ± 1.14%. After a certain period of drought treatment for seedlings, water supply was restored (85.00% ± 1.21%) until the plants matured. Three replicates were performed for each treatment. 

In a separate experiment, hydroponic experiments were conducted. The conditions of the climate chamber were 50–70% relative humidity, day/night temperature of 25/18 °C, and a photoperiod of 16 h light/8 h dark with approximately 300 μmol m^−2^ s^−1^ irradiation intensity. The seedlings were watered with 15% polyethylene glycol (PEG) 6000 contained in Hoagland solution for 21 d. The control medium did not have 15% PEG 6000 added. Fresh roots were used in root morphology and viability assays. The roots that were used in metabolomics studies and qRT-PCR (quantitative real-time polymerase chain reaction) were collected, frozen in liquid N_2_, and stored at −80 °C for analysis. Each treatment was made up of three biological replicates.

### 4.2. Agronomic and Quality Traits Analysis

Agronomic traits of two spring barley genotypes were measured after maturity, such as the plant height, spike length, biomass, the number of panicles per plant, effective tillers, and grains per panicle [43,44]. And 3 individual plants were randomly selected from each material during the mature period of barley. In addition, the thousand-grain weight, ash content, protein content, starch content, and cellulose content of barley seeds were determined using the Fox NIRS DA1650 near-infrared analyzer (FOSS, Hillerød, Denmark). 

### 4.3. Observation of Seed Morphology and Starch Granules

Seed morphology was discussed and photographed with a stereomicroscope to observe the grains’ appearances (Leica-M165 C; Leica, Wetzlar, Germany); 10 grains of barley per entry were taken for images with 24000dpi resolution. The different grain ultrastructures from various barley were investigated using scanning electron microscopy (SEM) according to Sangwongchai et al. description [45]. The mature seed of each treatment was cut longitudinally and mounted on double-sided thick adhesive tape metal stubs used for scanning electron microscopy (SEM). The seed longitudinal surface was scanned with a SEM (S-3400N, Hitachi Group Company, Tokyo, Japan) with 5.0 kV current and 60 Pa pressure. Each treatment had three biological replications.

### 4.4. Root Morphology and Root Viability Assay

Root morphology was determined by a WinRHIZO Program (Regent Instruments Inc., Saint-Foye, Canada). Drought stress tolerance of roots was measured by assessing relative roots viability. Relative root viability was measured using a 2,3,5-triphenyltetrazolium chloride reduction method [38]. About 0.2 g of fresh roots per sample were used. Each experiment was conducted three times.

### 4.5. Metabolite Extraction

The freeze-dried roots (80 mg) were grounded into a fine powder with a mortar and pestle. Methanol/acetonitrile/H_2_O (1000 μL; 2:2:1, v/v/v) was added to the homogenized solution for metabolite extraction. The mixture was centrifuged for 15 min (14,000× *g*, 4 °C). The supernatant was dried in a vacuum centrifuge (ShangHai, China, BILON-FD8OCE). For liquid chromatography–mass spectrometry analysis, the samples were redissolved in 100 μL acetonitrile/water (1:1, v/v) solvent. The quality control (QC) sample was developed by taking 20 µL from each metabolite extract and applied to detect the reproducibility of the samples from the same plant organ.

### 4.6. Metabolite Detection

Ultra performance liquid chromatography (UPLC) (Thermo, Waltham, MA, USA; UltiMate 3000) steps and tandem mass spectrometry (MS/MS; Thermo Q Exactive) analyses were conducted as previously described. In brief, 5 mL of each extracted sample was poured into an ACQUITY UPLC HSS T3 1.8 µm column (100 mm × 210 mm, 1.8 µm, Waters). In ESI positive mode, the mobile phase contained A = water with 0.1% formic acid and B = acetonitrile with 0.1% formic acid. In ESI negative mode, the mobile phase contained A = 0.5 mM ammonium fluoride in water and B = acetonitrile. The gradient was 1% B for 1.5 min and was linearly increased to 99% in 11.5 min and kept for 3.5 min. Then, it was reduced to 1% for 0.1 min, and 3.4 min of a re-equilibration period was employed. The gradients were at a flow rate of 0.3 mL/min, and the column temperatures were kept constant at 25 °C. A 2 µL aliquot of each sample was injected. The ESI source conditions were set as follows: Ion Source Gas1 as 60, Ion Source Gas2 as 60, curtain gas as 30, source temperature of 600 °C, IonSpray Voltage Floating (ISVF) ± 5500 V. In MS-only acquisition, the instrument was set to acquire over the m/z range 60–1000 Da, and the accumulation time for a time-of-flight mass spectrometry scan was set at 0.20 s/spectra. In auto MS/MS acquisition, the instrument was set to acquire over the m/z range 25–1000 Da, and the accumulation time for a product ion scan was set at 0.05 s/spectra. The product ion scan was acquired using information-dependent acquisition (IDA) with a high-sensitivity mode selected. The parameters were set as follows: the collision energy (CE) was fixed at 35 V with ±15 eV; declustering potential (DP) of 60 V (+) and −60 V (−); exclude isotopes within 4 Da, candidate ions to monitor per cycle were 10.

### 4.7. Metabolite Identification

Raw data files were transformed into a mzXML format through Protrowizard (v3.0.8789), and then data formatting was analyzed by the statistical package XCMS (v3.7.1). Peak identification, filtration, and alignment were conducted as previously described. Metabolites were employed by spectrum matching to databases: the Human Metabolome Database (HMDB) (http://www.hmdb.ca, accessed on 17 March 2024), Lipid Maps (http://www.lipidmaps.org, accessed on 17 March 2024), METLIN (http://metlin.scripps.edu, accessed on 17 March 2024), mzClound, and Massbank (http://www.massbank.jp/, accessed on 17 March 2024).

### 4.8. Statistical Analysis

In order to explore how the metabolic profiles differed between ZDM5430 and IL-12, the identified metabolites were used in principal component analysis (PCA), partial least squares discriminant analysis, and the orthogonal projection to latent structures discriminant analysis (OPLS-DA) using corresponding R package models (http://www.r-project.org/, accessed on 17 March 2024). The variable importance for the projection (VIP) score from the OPLS model was subjected to rank between the best-distinguished metabolites between varieties. Metabolites between two varieties with a VIP score ≥ 1 and *t*-test *p* < 0.05 were considered as a univariate analysis to determine the different metabolites, which mapped to Kyoto Encyclopedia of Genes and Genomes (KEGG) metabolic pathways for the enrichment analysis (FDR ≤ 0.05) of pathways. Hierarchical cluster analysis was executed on metabolite accumulation patterns between different varieties via a Pearson correlation distance matrix.

### 4.9. Real-Time Quantitative RT-PCR

Total RNA was isolated from the roots of different genotype barley, and cDNA was synthesized according to previous description [46]. The mRNA levels of the *UGP2*, *GAE*, *GPI*, *AXS*, *SS*, *SPS2*, *SS2*, *TPPJ*, *AT4G*, *BGLU12*, *INVA*, *BACOVA*, *TPS11*, and 18s genes were analyzed by ABI 7500 Real-Time PCR System in SYBR Green Master Mix (TaKaRa; Shanghai, China) with the primers listed in Table S11. The levels of genes expression were normalized against *Hordeum vulgare* L. 18S rRNA gene (an internal control). The relative gene expressions were observed based on the 2^−ΔΔCT^ method [47].

## 5. Conclusions

Our study highlighted the growth traits and differential metabolites in response to PEG-simulated drought stress between a drought-tolerant barley genotype, ZDM5430, and a drought-susceptible genotype, IL-12. The growth traits were different between two barley genotypes under different drought stresses. A total of 12,235 metabolites were identified. KEGG enrichment revealed that different metabolites were significant regarding enrichment in different cultivar roots under drought stress. The complete view of metabolite changes under PEG-simulated drought stress indicate that the TCA cycle, urea cycle, the met salvage pathway, amino acid metabolism, unsaturated fatty acid biosynthesis, phenolic metabolism, and glycolysis are involved in the response to drought stress, and a metabolic network in the roots of barley under drought was conducted. This study offers an improved insight on the metabolite response to drought stress in barley roots, and it lays the foundation for breeding drought-tolerant barley genotypes.

## Figures and Tables

**Table 1:** The size of grain in ZDM5430 and IL-12 under different drought stress conditions. Data are shown as mean ± standard error (n = 3). ANOVA: Since two-way ANOVA revealed a significant ozone effect on PII values according to Tukey’s post hoc test, different letters indicate significant differences among means in each group (*p* ≤ 0.05).

Cultivars	Treatment	Length (cm)	Width (cm)	Width-to-Length Ratio	Volume (cm^3^)
ZDM5430	CK	1.41 ± 0.01 a	0.73 ± 0.02 b	0.52 ± 0.03 d	0.21 ± 0.01 ab
	T1	1.32 ± 0.02 b	0.65 ± 0.01 h	0.49 ± 0.03 e	0.17 ± 0.02 cd
	T2	1.31 ± 0.02 bc	0.72 ± 0.02 c	0.55 ± 0.02 c	0.19 ± 0.01 bc
	T3	1.21 ± 0.01 f	0.68 ± 0.01 e	0.56 ± 0.02 bc	0.16 ± 0.01 d
	T4	1.21 ± 0.03 f	0.70 ± 0.02 d	0.58 ± 0.01 ab	0.17 ± 0.02 cd
	T5	1.09 ± 0.01 ij	0.63 ± 0.01 i	0.58 ± 0.02 ab	0.14 ± 0.01 e
IL-12	CK	1.30 ± 0.01 c	0.74 ± 0.01 a	0.57 ± 0.01 b	0.22 ± 0.01 a
	T1	1.24 ± 0.02 d	0.73 ± 0.02 b	0.59 ± 0.01 a	0.21 ± 0.02 a
	T2	1.23 ± 0.01 de	0.70 ± 0.01 d	0.57 ± 0.02 b	0.20 ± 0.01 b
	T3	1.18 ± 0.01 h	0.67 ± 0.02 f	0.57 ± 0.03 b	0.18 ± 0.01 c
	T4	1.20 ± 0.01 fg	0.66 ± 0.01 g	0.55 ± 0.02 c	0.18 ± 0.02 c
	T5	1.10 ± 0.02 i	0.60 ± 0.02 j	0.55 ± 0.01 c	0.15 ± 0.02 de

## Data Availability

Data are contained within the article and Supplementary Materials.

## Supplementary Materials

The following supporting information can be downloaded at: https://www.mdpi.com/article/10.3390/ijms25137224/s1.
