# Supplementary material for: Growth Properties and Metabolomic Analysis Provide Insight into Drought Tolerance in Barley (Hordeum vulgare L.)
Source: Int J Mol Sci. 2024 Jun 29;25(13):7224. doi: 10.3390/ijms25137224 (PMC11241679; doi:10.3390/ijms25137224)
Supplement: Supplementary file 1 [file ijms-25-07224-s001.zip › supplementary table S11.pdf]

Table S11 Primers used in this study

Gene	Primer sequence (5′-3′)	Annealing temperature/℃
Actin	F: AAGCAGCCAGAATGTACAGCGAGAAC<br>R: GGTACAGACCAGCAAAGCCAGAAATG	80
UGP2	F:CGTGGCGAACTCAGACAACTTGG<br>R:GCTCATCAGGGACTTGGGCAATC	81
GAE	F:CGGGTGAAGGCGAAGAAGCA<br>R:GGTGTAGCCGTAGTAGGAGAGGTA	77
GPI	F:GCTTCTTCTGCTCGTGCTCCTC<br>R:TCCGCTCGTCAGGTATTCCTAATCT	80
AXS	F:TCCTTCCACCGCCTCAACATCA<br>R:GATCAGACGCTTGCCGTTCTCC	81
SS	F:TGCGAGCGAGACTATCCACCTT<br>R:CTGAGCGAAGTAACCATGAGGAGAG	80
SPS2	F:GTTCTCACTGGTCACTCGCTTGG<br>R:CTGCTCATCAATCTCCTGCCTTGT	79
SS2	F:GCTCCAAGACCTTCTGCCGATTC<br>R:CTGCTCTGTTGCGTCACCACTG	78
TPPJ	F:ACTGAGTTCCTGCCTGTCATCCA<br>R:CAGAGTTCCTTGTAGCCGTCCAG	81
AT4G	F:GTTCATAGAAGGCGAGGCGTTGT<br>R:AGGTACTTGGCGTAGGTCAGGAG	80
BGLU12	F:TCAAGGTCTCCGTGATCTGCTACT<br>R:GGTGCCTGTGGTGGTAATCTATCC	81
INVA	F:AGCGTCTCTGGCACTGGCAT<br>R:CTTGTTGGCAGCAGCGTCGTA	79
BACOVA	F:AGTCGCTGGTGCTCCTGAAGAA<br>R:GTCGCCTTGCCACTTGATCGT	78
TPS11	F:GCGGAGTGCGTCGTTGTTACTG<br>R:ACTCAATGATGGCGAGCAACCTATG	80
