# Supplementary figures and images for: Growth Properties and Metabolomic Analysis Provide Insight into Drought Tolerance in Barley (Hordeum vulgare L.)
Source: Int J Mol Sci. 2024 Jun 29;25(13):7224. doi: 10.3390/ijms25137224 (PMC11241679; doi:10.3390/ijms25137224)

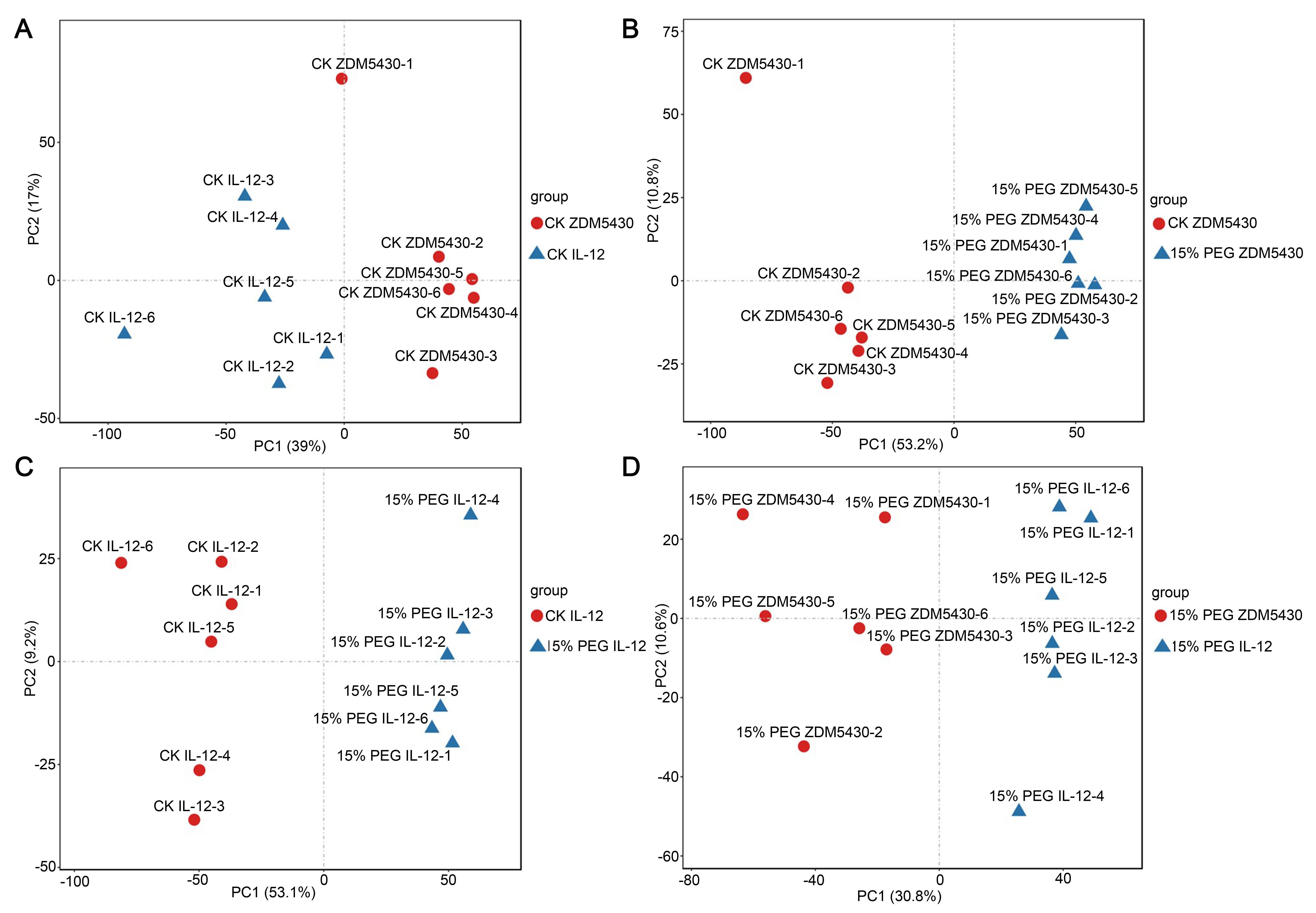

Supplement: Supplementary file 1 [file ijms-25-07224-s001.zip › Supplementary figure S1.jpg]

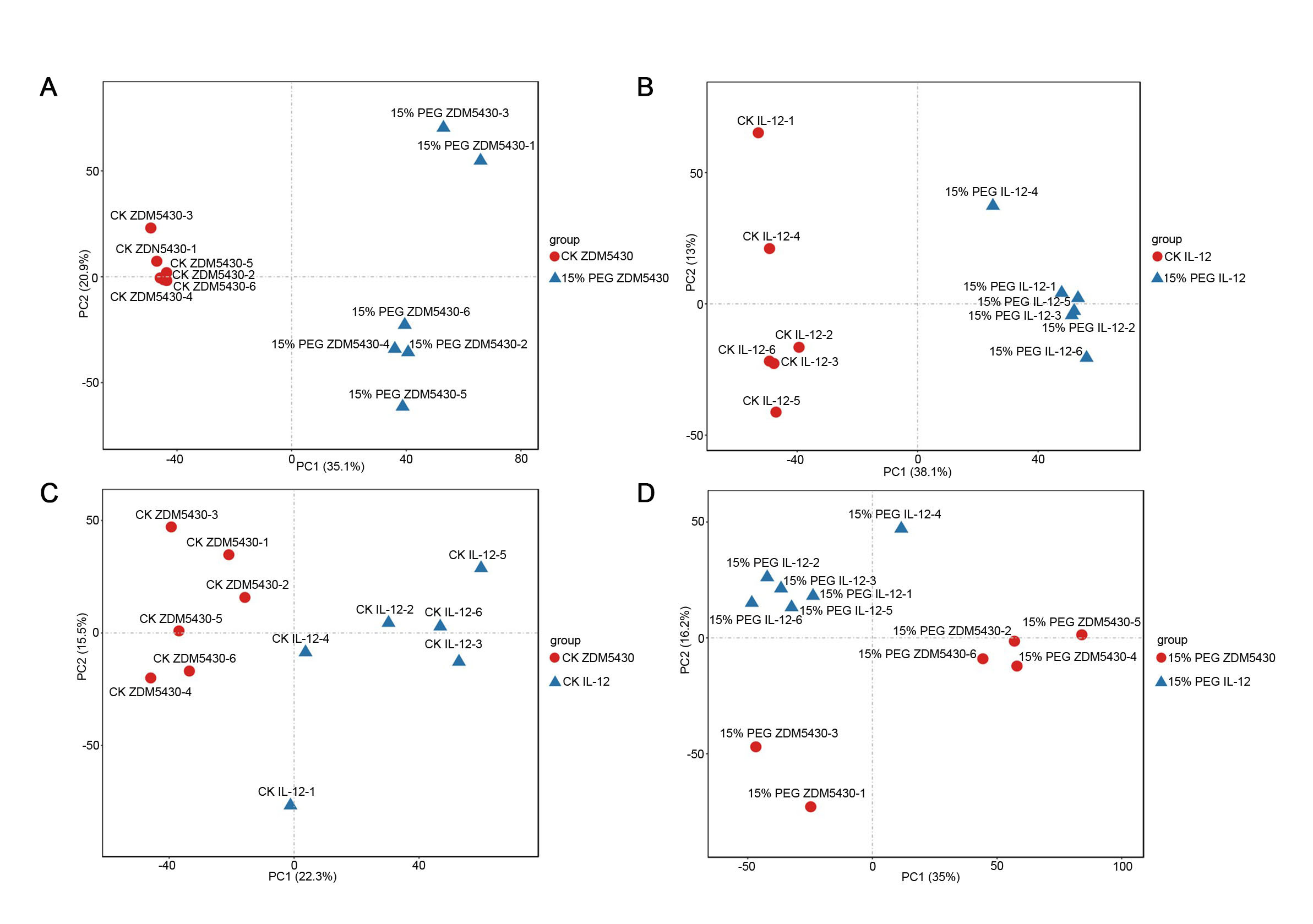

Supplement: Supplementary file 1 [file ijms-25-07224-s001.zip › Supplementary figure S2.jpg]

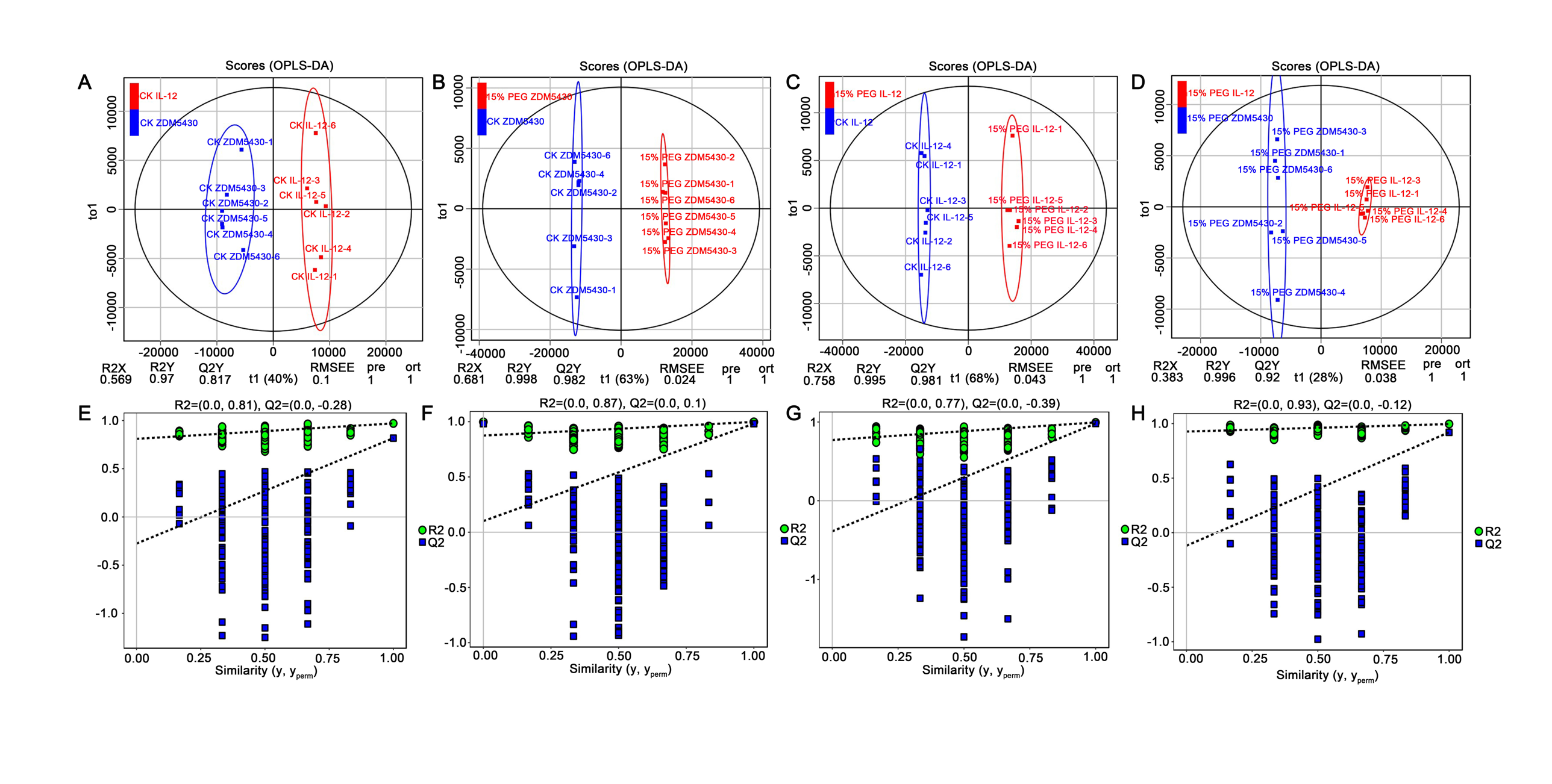

Supplement: Supplementary file 1 [file ijms-25-07224-s001.zip › Supplementary figure S3.jpg]
